# Molecular Pathological Classification of Neurodegenerative Diseases: Turning towards Precision Medicine

**DOI:** 10.3390/ijms17020189

**Published:** 2016-02-02

**Authors:** Gabor G. Kovacs

**Affiliations:** Institute of Neurology, Medical University of Vienna, AKH 4J, Währinger Gürtel 18-20, A-1090 Vienna, Austria; gabor.kovacs@meduniwien.ac.at; Tel.: +43-1-40400-55070; Fax: +43-1-40400-55110

**Keywords:** biomarker, classification, molecular pathology, neurodegenerative disease, proteinopathy

## Abstract

Neurodegenerative diseases (NDDs) are characterized by selective dysfunction and loss of neurons associated with pathologically altered proteins that deposit in the human brain but also in peripheral organs. These proteins and their biochemical modifications can be potentially targeted for therapy or used as biomarkers. Despite a plethora of modifications demonstrated for different neurodegeneration-related proteins, such as amyloid-β, prion protein, tau, α-synuclein, TAR DNA-binding protein 43 (TDP-43), or fused in sarcoma protein (FUS), molecular classification of NDDs relies on detailed morphological evaluation of protein deposits, their distribution in the brain, and their correlation to clinical symptoms together with specific genetic alterations. A further facet of the neuropathology-based classification is the fact that many protein deposits show a hierarchical involvement of brain regions. This has been shown for Alzheimer and Parkinson disease and some forms of tauopathies and TDP-43 proteinopathies. The present paper aims to summarize current molecular classification of NDDs, focusing on the most relevant biochemical and morphological aspects. Since the combination of proteinopathies is frequent, definition of novel clusters of patients with NDDs needs to be considered in the era of precision medicine. Optimally, neuropathological categorizing of NDDs should be translated into *in vivo* detectable biomarkers to support better prediction of prognosis and stratification of patients for therapy trials.

## 1. Introduction

Neurodegenerative diseases (NDDs) are characterized by progressive dysfunction and loss of neurons leading to distinct involvement of functional systems defining clinical presentations. Revolutionary studies demonstrated that proteins with altered physicochemical properties deposit in the human brain as a fundamental phenomenon in most NDDs, defined also as conformational diseases [1]. The pathological conformers are also called misfolded proteins. Placing proteins in the center of the pathogenesis of NDDs led to further discoveries, such as uncovering the role of the unfolded protein response [2]. In addition, it has been recognized that protein elimination pathways, like the ubiquitin-proteasome system and the autophagy-lysosome pathway [3], stress response proteins and chaperones have high impact on the pathogenesis. These pathways interact with other pathways such as those of energetic dysregulation, molecular damage, metabolic changes, dysregulation of ion homeostasis, and adaptation [4]. Indeed, there are several pathways, which contribute to the damage of neurons. For example, the role of l-glutamate (or l-aspartate) mediated acute excitotoxicity in cerebral ischemia or status epilepticus is well known. However, chronic excitotoxicity has been discussed for progressive long-term neurodegenerative processes, including amyotrophic lateral sclerosis (ALS), Alzheimer disease (AD), and Huntington disease (HD); even if the molecular basis for this varies widely and might be distinct for each disease [5]. On the other hand, neurons are exhausted by converging pathways of altered mitochondria and energy metabolism, voltage-dependent anion channel, and lipid rafts as exemplified in AD [6]; furthermore, mitochondrial dysfunction is also central in the pathogenesis of Parkinson disease (PD) [7]. The intimate relation between microglial activation, nitric oxide and neuroinflammation in the human brain is also widely discussed in relation to NDDs [8]. Oxidative stress and formation of free radicals/reactive oxygen species, mitochondrial dysfunctions, impaired bioenergetics and DNA damage, neuroinflammatory processes and disruption of cellular/axonal transport are linked to the formation of toxic forms of NDD-related proteins [9]. These pathways most likely do not represent the “only pathways” leading to neurodegeneration; however, they are interrelated in a complex way leading to neuronal dysfunction and death. Better stratification of diseases would be required to understand which of these predominates in different conditions. This underpins the importance of molecular classification of NDDs also for fine-tuning our knowledge on pathogenic pathways. 

The central role of proteins has been translated into biomarker research and also into the development of novel therapeutic strategies. Indeed, vaccination against α-synuclein, amyloid-β (Aβ), or tau has been explored, in particular that these proteins seem to propagate cell-to-cell and may be accessible to antibodies [10]. Disease-modifying therapeutic strategies may require reducing the synthesis, preventing the aggregation and/or enhancing the clearance of the pathological forms of proteins [10]. These aspects also emphasize the importance of protein-based classification of NDDs and its translation into *in vivo* biomarkers capable of detecting diseases as early as possible. Protein-based biomarkers would be needed for the stratification of patients for anti-protein therapies, in particular since many of the NDDs show overlapping clinical features and also combined deposition of proteins.

Widespread application of specific antibodies against NDD-related proteins and their modifications led to an explosion of descriptions of novel neuropathological phenotypes and enabled the development of reliable diagnostic criteria. Many disorders are associated with the degeneration of neurons, including immunological disorders; furthermore, several gene alterations lead to the dysfunction of the encoded proteins. However, not all of these processes associate with microscopically detectable protein depositions, at least not with the currently applied techniques. For example, in hereditary spastic paraplegia, the neuropathological examination, without knowledge of the clinical symptoms, can suggest the condition but there are no specific protein inclusions that allow the observer to link the pathology to a specific gene mutation. Indeed, only rare reports describe TDP-43, tau or crystalloid deposits in hereditary spastic paraplegia [11,12,13], but their detection is not enough to suggest the gene involved in the development of the disease. Similarly, there are many different mutations, which are associated with spinocerebellar ataxia (SCA) [14], but not all of them show protein inclusions, emphasizing the diverse pathogenesis. This review aims to summarize current concepts of disease classification with a focus on the molecular pathological aspects of those neurodegenerative conditions of the adulthood where microscopically detectable protein deposits have been described (”neurodegenerative proteinopathies”). There are a considerable number of possible combinations of proteinopathies, also referred to in the frame of mixed pathologies [15]; detailed discussion of these is out of the scope of the present paper.

## 2. Concepts of Disease Classification

Classification of NDDs is based on the following [16,17]:

Major clinical symptoms: these are determined by the anatomical region showing neuronal dysfunction or loss and do not necessarily reflect the molecular changes in the background.

Proteins that show conformational change and biochemical modifications.

Cellular and subcellular pathology: this means whether neurons or glial cells (either or both astro- and oligodendroglia), including which compartment of the cells, show pathological protein deposits; or whether these are found extracellularly.

**Figure 1:**
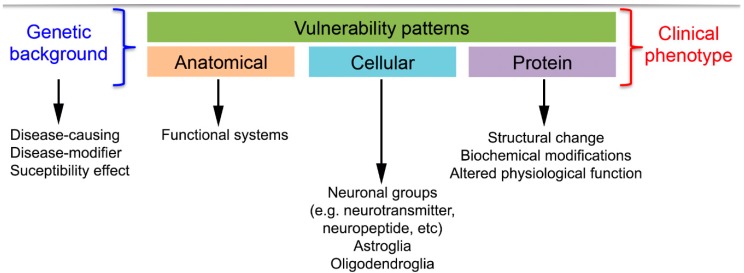
Summary of the concept of vulnerability patterns in neurodegenerative diseases. Coloured boxes represent different vulnerability patterns.

Accordingly, in association with a neurodegenerative syndrome one can define anatomical, cellular and protein vulnerability (Figure 1) [18]. Genetic alterations can also lead to these alterations or influence the susceptibility to develop these diseases.

### 2.1. Anatomical Involvement of Neuronal Loss Underlying Clinical Symptomatology 

In most cases, clinical symptoms overlap during the course of the disease. Thus, clinical classification is helpful for focusing on the early symptoms. Cognitive decline, dementia, and alterations in high-order brain functions are associated with involvement of the entorhinal cortex, hippocampus, limbic system and neocortical areas. A subtype of dementia is frontotemporal dementia (FTD), which is associated with degeneration of the frontal and temporal lobes (frontotemporal lobar degeneration, FTLD). In movement disorders the basal ganglia, thalamus, brainstem nuclei, cerebellar cortex and nuclei, motor cortical areas and lower motor neurons of the spinal cord are involved. Combinations of these symptoms are observed in some diseases (*i.e.*, prion diseases) early during the clinical course and in many disorders during the progression. 

### 2.2. Neuropathological-Biochemical Classification 

This focuses primarily on the evaluation of the anatomical distribution of neuronal loss, and additional histological features (e.g., vascular lesions or spongiform change of the neuropil), and the distinction of intracellular and extracellular protein accumulations, which are analyzed by immunohistochemistry complemented by biochemistry. There are some aspects, which need to be clarified to understand the neuropathological approach. Firstly, not all protein-deposits visible by immunohistochemical methods show the amyloid staining property (means that a particular structure shows apple-green birefringence under polarized light when stained with the Congo red dye) even though they are composed of proteins with highly ordered stacks of β-sheet-rich elements. Secondly, for some proteins, synaptic location is mentioned; however, except in the case of prion disease (see below), this is not respected for diagnostic classifications. It is important to distinguish the subcellular location of the intracellular deposits; whether they are nuclear, cytoplasmic, or neuritic (axonal or dendrites), or in cellular processes (*i.e.*, for astrocytes). For some diseases, only morphological criteria are used for subtyping, whereas for others biochemical modifications or even a gene polymorphism are also considered. For all neurodegenerative proteinopathies there are hereditary forms described.

## 3. Altered Proteins in Neurodegenerative Diseases 

The following proteins are mostly associated with NDDs [19]: (1) The microtubule-associated protein Tau, which is encoded by a single gene (*MAPT*) on chromosome 17q21; (2) Amyloid-β (Aβ), which is cleaved from a large transmembrane precursor protein (Aβ-precursor protein or AβPP). encoded by the *AβPP* gene on chromosome 21q21.3; (3) α-Synuclein, which is encoded by a gene (*SNCA*) on chromosome 4; (4) Prion protein (PrP), which is encoded by a gene (*PRNP*) located to chromosome 20; (5) Transactive response (TAR) DNA-binding protein 43 (TDP-43), which is a highly conserved nuclear protein [20], encoded by the *TARDBP* gene on chromosome 1; (6) Fused in sarcoma protein (FUS), Ewing’s sarcoma RNA-binding protein 1 (EWSR1), and TATA-binding protein-associated factor 15 (TAF15), collectively called as FET proteins [21]. The most examined is FUS, which is a 526 amino acid long protein encoded by a gene on chromosome 16; (7) Further proteins are associated mostly with hereditary disorders; these comprise proteins encoded by genes linked to neurological trinucleotide repeat disorders (e.g., huntingtin, ataxins, atrophin-1), neuroserpinopathy, ferritin-related NDDs, and familial cerebral amyloidoses. 

Based on the major protein showing depositions in the central nervous system, neurodegenerative proteinopathies are classified as tauopathies, α-synucleinopathies, TDP-43 proteinopathies, FUS/FET proteinopathies, prion diseases, trinucleotide repeat diseases, neuroserpinopathy, ferritinopathy, and cerebral amyloidoses (Table 1). The nomenclature of proteinopathies overlap with clinicopathological descriptions, like the grouping of FTLD as FTLD-tau, FTLD-TDP, FTLD-FUS, FTLD-UPS (when profiles immunoreactive only for the ubiquitin-proteasome system are detected) or FTLD-ni (when no specific inclusions are detected); or SCA for a group of trinucleotide repeat diseases. 

Despite the plethora of biochemical modifications of these proteins (Table 2) (for reviews see: [19,22,23,24,25,26,27,28,29,30,31,32,33]), only a small number are currently implemented in molecular pathologic subtyping or for biomarker development. For example, molecular classification of human prion disease is based on the Western blot pattern of PrP^res^ (*i.e.*, PK-resistant PrP) together with the codon 129 polymorphism of the *PRNP* [34,35]. For Aβ, biochemical modifications are not included in the classification of AD. However, the observation of the existence of different biochemical stages of Aβ aggregate maturation and that different variants of Aβ peptides may result from alternative processing or from mutations that lead to rare forms of familial AD, have implications for understanding early and late phases of AD with relevance to the characterization and interpretation of AD-type pathology [27]. Tauopathies are distinguished based on the ratio of three repeat (R)- and 4R-tau and two or three major phospho-tau bands (60, 64, and 68 kDa) in Western blot of sarkosyl-insoluble fractions [25,36,37]. Differences in phosphorylation of tau epitopes can be also helpful biochemical markers. For example, evaluation of neurofibrillary tangle-related pathology suggested that phosphorylation of tau protein at the carboxyl terminus may be among the earliest tau events [38]. A recent study attempted a biochemical classification of tauopathies by immunoblot, protein sequence and mass spectrometric analyses of sarkosyl-insoluble and trypsin-resistant tau [39]. It has been shown that the banding patterns of C-terminal fragments of tau are different among different tauopathies, and the trypsin-resistant band patterns are also distinct [39]. Phosphorylation at specific epitopes is important also in α-synucleinopathies and TDP-43 proteinopathies; however, they are not sufficient to distinguish currently defined disease types. However, different banding patterns of C-terminal fragments of phosphorylated TDP-43 seem to differentiate representatives of multisystem TDP-43 proteinopathies [40]. FUS was detected in the SDS soluble fractions of a subset of FTLD cases, however these immunoblot examinations did not identify any specific signals, which would have relevance for subtyping [41]. 

## 4. Molecular Pathological Subtyping 

For the neuropathological classification a few of these biochemical aspects are considered; however, currently the most important step is to evaluate the localization and distribution of proteins [42]:

Extracellular deposits comprise deposits with immunoreactivity for Aβ or PrP. Importantly, disease-associated PrP shows a synaptic pattern of deposition.

Major proteins that deposit intracellularly include tau, α-synuclein, TDP-43, FUS/FET proteins, and those associated with trinucleotide repeat disorders or rare hereditary diseases. 

**Table 1:** Summary of most relevant modifications of neurodegeneration related proteins with remarks.

Disease Group	Protein	Disease Type	Form	Phenotype
AD	Tau, Aβ	AD	SP/GEN	DEM
Tauopathy (FTLD-Tau *)	Tau	PiD	SP	FTD
		GGT	SP	FTD
		CBD	SP	MD/FTD
		PSP	SP	MD/FTD
		AGD	SP	DEM
		NFT-dementia/PART	SP	DEM
		FTDP-17T	GEN	FTD/MD
TDP-43 proteinopathy	TDP-43	FTLD-TDP (type A–D)	SP/GEN	FTD
		MND-TDP	SP/GEN	MD
		FTLD-MND-TDP	SP/GEN	FTD-MD
FUS (FET)-proteinopathy FTLD/MND-FUS (FET)	FUS/FET	aFTLD-U, NIFID, BIBD	SP	FTD/MD
		MND-FUS	GEN	MD
α-Synucleinopathy	α-Synuclein	PD	SP/GEN	MD
		DLB	SP/GEN	DEM/MD
		MSA	SP	MD
Prion disease	PrP	sCJD, VPSPr, sFI	SP	DEM/MD
		iCJD	ACQ	DEM/MD
		vCJD	ACQ	DEM/MD
		Kuru	ACQ	DEM/MD
		gCJD, GSS, FFI, PrP-CAA	GEN	DEM/MD
TRD **	Huntingtin	HD	GEN	MD
	Ataxin 1, 2, 3, 7, CACNA1A, TBP	SCA 1, 2, 3, 6, 7, 17	GEN	MD
	FMRP	FXTAS	GEN	MD
	ARP	SBMA	GEN	MD
	Atrophin-1	DRPLA	GEN	MD
Other forms	Ferritin	Hereditary ferritinopathy	GEN	DEM/MD
	Neuroserpin	Neuroserpinopathy	GEN	DEM
	ABri, ADan, gelsolin, cystatin, transthyretin, Aβ	Hereditary amyloidoses/CAA	GEN	DEM
	Only UPS	FTLD-UPS	GEN	FTD
	Not determined	FTLD-ni	SP	FTD
	Tau, α-Synuclein	NBIA	GEN	DEM/MD
	Tau, a-Synuclein, TDP-43	Various genetic and sporadic diseases (“secondary” proteinopathy forms)	SP/GEN	DEM/MD

Abbreviations: ACQ: acquired; ARP: androgen receptor protein; AD: Alzheimer disease; AGD: Argyrophilic grain disease; BIBD: Basophilic inclusion body disease; CAA: sporadic cerebral amyloid angiopathy; CACNA1A: α1A subunit of the P/Q-type voltage-gated calcium channel (SCA6; Cytoplasmic aggregates); CBD: Corticobasal degeneration; CJD: Creutzfeldt-Jakob disease (i: iatrogenic, s: sporadic, v: variant, g: genetic); DEM: dementia; DLB: Dementia with Lewy bodies; DRPLA: dentatorubral-pallidoluysian atrophy; sFI: sporadic fatal insomnia; FFI: fatal familial insomnia; FMRP: Fragile X mental retardation protein; FTD: frontotemporal dementia; FTLD: frontotemporal lobar degeneration; aFTLD-U: atypical FTLD with ubiquitinated inclusions; FTLD-UPS: FTLD with inclusions immunoreactive only for the components of the ubiquitine proteasome system; FTLD-ni: FTLD no inclusion specified; FTDP-17T: Frontotemporal dementia and parkinsonism linked to chromosome 17 caused by mutations in the *MAPT* (tau) gene; FXTAS: Fragile X associated tremor and ataxia syndrome (here also astroglial inclusions); GEN: genetic; GGT: globular glial tauopathy; GSS: Gerstmann-Sträussler-Scheinker disease; HD: Huntington disease; INIBD: intranuclear inclusion body diseases; MD: movement disorder; MND: Motor neuron disease; MSA: multiple system atrophy; NBIA: neurodegeneration with brain iron accumulation; NIFID: Neurofilament intermediate filament inclusion disease; PD: Parkinson disease; PiD: Pick disease; PrP: prion protein; PSP: Progressive supranuclear palsy; SCA: spinocerebellar ataxia; SBMA: spinal and bulbar muscular atrophy; SP: sporadic; TBP: TATA-Box binding protein (SCA17); TRD: trinucleotide repeat expansion disorder: refers to genetic disorder and associated with different proteins; VPSPr: variably protease-sensitive prionopathy. * FTLD is not typical in AGD or PART; ** only SCAs with protein inclusions.

**Table 2:** Neurodegeneration-related proteins and their modifications with potential relevance for disease classification (summarized from references [21,22,23,24,25,26,27,28,29,30,31,32,33,34,35,36,37,38,39,40,41]).

Protein	Remarks on Modifications with Potential Relevance for Classification
Aβ	Aβ peptides are produced by the sequential cleavage by different proteases (e.g., β- followed by γ-secretases)
	Aβ 1-40/1-42 peptides are the most abundant components of Aβ deposits
	N-terminal truncation of soluble and insoluble Aβ peptide species as well as C-terminally truncated Aβ species (1-37/38/39) have been also described
	Aβ deposits may have distinct PK resistance
	Further aspects: pyroglutamate modifications at residues 3 or 11 (AβN3pE and AβN11pE); isomerization/racemization (D-Asp or L-isoAsp at N1, N7); glycosylation; phosphorylation at Serine residue 8 and 26 (pSer8Aβ and pSer26Aβ)
PrP	The physiological cellular form of PrP (PrPC) is a detergent soluble, PK sensitive protein that has endogenously truncated fragments, while the disease-associated PrPSc is detergent-insoluble and resistant to PK treatment (termed PrPres)
	Based on differences in electrophoretic mobility and N-terminal sequence of the core fragments, different forms of PrPres were distinguished. The most common PrPres species is PrP27-30. Further fragments are for example PrP 11, PrP7-8, PrP14, PrP-CTF12/13, PrP16-17, and PrP17.5-18
	Oligomer form of PrP has been also described
Tau	Alternative splicing generates six isoforms, which are present in the adult human brain; based on the absence or presence of exon 10 tau isoforms, either three or four repeat (3R, 4R) domains are distinguished
	Hyperphosphorylation is a common modification
	Tauopathies are distinguished based on the ratio of 3R- and 4R-tau and two or three major phospho-tau bands (60, 64, and 68 kDa) in Western blot of sarkosyl-insoluble fractions
	Further aspects: N- and C-terminal truncation, glycosylation, glycation, nitration of tyrosine residues, transglutamination, deamidation; acetylation; oligomer; the banding patterns of C-terminal fragments of tau and the trypsin-resistant band patterns are distinct among tauopathies
a-Syn	Phosphorylation at serine 87 and 129 (most relevant) and at tyrosine 125 residue
	Further aspects: Nitration, glycosylation, C-terminally truncated species; oligomer forms (also physiological native oligomers called multimers: α-synuclein exists in various conformations and oligomeric states in a dynamic equilibrium); PK-resistant form is also described
TDP-43	Phosphorylation on serine 379 (S379), S403, S404, S409, S410 residues
	Further aspects: ubiquitinylation and abnormal cleavage; oligomer; C-terminal fragments detected in disease
FUS	FUS was detected in the SDS soluble fractions of a subset of FTLD cases

A further facet of the neuropathology-based classification is the fact that many protein deposits show a hierarchical involvement of brain regions. This is also referred to in the frame of prion-like spreading. It must be noted that in prion disease staging of protein deposition is not established. The concept of prion-like spreading stems from observations that prion disease-type pathology and PrP deposits spread within the CNS via central axons of specific pathways after experimental inoculation [43,44]. Recent findings suggest disease-associated protein seeds as essential components in the initiation and expansion of aggregated proteins in diverse NDDs [45], although human-to-human transmission of the full phenotype of a disease has, at present, only been proven for prion diseases. Studies in humans revealed particular pathways by which disease-specific protein pathology might spread through the CNS complementing experimental evidences (cell culture and animal models) of the molecular mechanisms of protein propagation in AD (involving tau and Aβ), FTLD-tau (involving tau), PD (involving α-synuclein), ALS and FTLD-TDP (both involving TDP-43) [46]. Furthermore, seeded aggregation and cell-to-cell spreading in cell culture models have been demonstrated for mutated proteins of hereditary disorders (e.g., huntingtin, ataxins, and superoxide dismutase 1, SOD1) [47]. In addition to providing a basis for developing therapeutical strategies that halt the spread of protein deposits, this concept has important implications for classification, biomarker research, and clinicopathological correlation, since early and late stages or phases of certain diseases can be distinguished.

### 4.1. Alzheimer Disease

#### 4.1.1. Overview of Neuropathological Features

AD is characterized by the extracellular deposition of Aβ fibrils and by the intraneuronal accumulation of abnormally phosphorylated tau protein. Preclinical AD can be discussed * in vivo* based on the presence of biomarkers [48,49] or *post mortem* by the presence of AD-type neuropathological alterations despite no signs of cognitive decline during life. For tau pathology only neuronal tau immunoreactivities are considered (neuropil threads, neurofibrillary tangles and pretangles). Aβ-fibrils can deposit in the parenchyma in the form of plaques and in the vessel walls as cerebral amyloid angiopathy (CAA). Parenchymal Aβ deposits show different morphologies, like stellate (probably related to astrocytes), diffuse deposits (further divided into fleecy, lake-like or subpial) or focal deposits (such as those with or without a dense core), and further rare morphologies like cotton-wool plaques [23]. Distinct plaque types are related mostly to their anatomical distribution [27]. There are studies, which describe intraneuronal Aβ deposits with relevance for the understanding of the pathogenesis [50,51,52,53,54,55]. The differences in antibodies and pretreatments used hinder the harmonized evaluation of intracellular Aβ, and therefore it is not included in classification of AD or for defining any subtypes. Furthermore, there are no ubiquitinated intracellular Aβ inclusion bodies described in AD as there have been for tau, α-synuclein, TDP-43, FUS, and even for PrP [56]. The significance of astrocytic Aβ also awaits clarification [23].

#### 4.1.2. Aspects of Classification

For the classification and diagnosis of AD, the stages of neurofibrillary degeneration and the phases of Aβ deposition should be documented. Protein pathology in AD often follows a stereotypical pattern that was conceptualized by Braak and coworkers, either as a staging scheme for neurofibrillary (tau) pathology [57,58] or as phases for Aβ deposition [59]. Regarding tau pathology, strategic areas include the transentorhinal cortex (stage I), entorhinal cortex (stage II), inferior (stage III) and middle temporal gyri (stage IV), while for the end stages an examination of the occipital cortex is needed (peristriate for stage V and striate cortex for stage VI) [57,58,60]. Further studies showed that subcortical nuclei also show early tau pathology [61,62], indicated also as early subcortical stages a, b, c [63,64], or as early pre-cortical evolutional phase [65]. Regarding Aβ deposition, five phases were proposed by the progressive involvement of isocortical areas (phase 1), hippocampus and entorhinal cortex (phase 2), basal ganglia and diencephalon (phase 3), brainstem (phase 4) and cerebellum (phase 5). This is frequently but not inevitably accompanied by CAA distinguished as capillary (type 1) or non-capillary type (type 2), also showing 3 stages [66,67]. Together with the classical semiquantitative scoring of neuritic plaques [68], the Braak stages and Thal phases are included in the recent NIA-AA neuropathological criteria for AD [69]. It must be noted, however, that using thioflavin-S fluorescence microscopy for the assessment of the distribution of neurofibrillary tangles led to the proposal of clinicopathological subtypes of AD, e.g., hippocampal sparing, typical, or limbic predominant [70]. Another study suggested tangle intensive and plaque intensive cases [71]. These studies argue for further subclassification of AD. On the other hand, it must be emphasized that both phosphorylated tau and Aβ can be seen in healthy young individuals [63,64,72,73], so the boarders between diseased and non-diseased or pre-diseased should be defined more precisely.

In summary, for the current classification of AD, phases of Aβ deposition, including the appearance of neuritic plaques, and stages of neurofibrillary degeneration (tau pathology) have to be documented together with the presence and stages of different types of CAA. Currently, biochemical markers (summarized in Table 2) are not yet implemented in further subtyping of AD.

### 4.2. Prion Diseases

#### 4.2.1. Overview of Neuropathological Features

Immunohistochemistry for disease-associated PrP is important for the neuropathological (definite) diagnosis. Due to the lack of commercially available conformational antibodies, special pre-treatments, such as Proteinase-K (PK), formic acid, and autoclaving are required to abolish detectability of the physiological PrP^C^ [74]. Major disease-associated PrP deposits comprise diffuse/synaptic, fine or coarse perineuronal immunopositivity, patchy/perivacuolar, plaque-like (lacking amyloid characteristics, thus called PrP plaques), and amyloid plaques [24]. In idiopathic (or sporadic) form of Creutzfeldt-Jakob disease (CJD) these deposits correlate well with the molecular phenotype, which is based on biochemical examination of PrP^res^ (PK-resistant PrP) and the evaluation of codon 129 of the *PRNP* [34,35,75]. In the bovine spongiform encephalopathy (BSE)-related variant CJD further morphologies can be detected, such as the characteristic florid plaques, *i.e.*, amyloid plaques surrounded by a collar of vacuoles and additional PrP immunoreactivities such as multiple small cluster plaques, amorphous and star-like pericellular and perivascular PrP deposits [76]. These are thought to be unique, although florid plaque-like structures have been described in iatrogenic CJD [77]. Peripheral organs also show accumulation of disease-associated PrP in humans, depending on the aetiological subtype [78,79,80,81,82,83,84]; some of these can be used for the diagnostic practice (e.g., lymphoreticular tissue in variant CJD [85]). Vessel wall deposition without amyloid angiopathy has been described in variant and sporadic CJD [86]. In certain genetic prion diseases CAA has been described [87]. In the recently reported disorder named variably proteinase sensitive prionopathy (VPSPr) the characteristic morphologies are target-like PrP deposits in a focal or more diffuse background of punctate or synaptic staining in the cerebrum, furthermore dot-like and small plaque-like deposits [88]. In Gerstmann-Sträussler-Scheinker disease (GSS) amyloid plaques, including those with multicentric cores, are highly specific findings [87]. Fatal familial insomnia (FFI) is characterized by a specific mutation (*PRNP * D178M-codon 129M) together with selective thalamic degeneration (seen also in sporadic fatal insomnia), although PrP deposits can be detected in other brain regions [89,90,91]. These disorders are associated with specific Western blot pattern of PrP^res^. In a cohort of cases with selective thalamic degeneration, we described peculiar intraneuritic distribution in neocortical regions, despite the lack of detectable PK-resistant PrP [92]. 

Although extracellular and presynaptic PrP deposits predominate the morphologies detected by PrP immunostaining, intraneuronal PrP immunoreactivities can be also observed. In a recent systematic study, we distinguished four types of neuronal PrP immunopositivities [56]. One of these (type II) has been shown to associate with the endosomal-lysosomal system (ELS). Interestingly, similarly to CJD brains [93], endosomes and lysosomes are enlarged and play an important role in the pathogenesis of AD [94]. On the other hand, another type (type IV) of intraneuronal PrP deposit seen in genetic CJD (E200K) showed ubiquitin immunoreactivity and has been localized ultrastucturally in aggresome-like structures and in autophagic vacuoles [56]. This contrasts with AD, where these have not been demonstrated. The presence of intraneuronal PrP immunoreactivity within endosomes and lysosomes support ultrastructural findings in animals and experimental models of prion diseases [95,96,97,98,99,100] and suggest that this pathway is one important way for the neuronal uptake of pathological proteins, *i.e.*, of the “prion-like spreading”. 

Finally, it has to be noted that in addition to immunohistochemistry, a special method, paraffin-embedded-tissue blot (PET-blot) where tissue sections are placed on a nitrocellulose membrane, and digested with PK leading to the blotting of the disease-associated PrP on the membrane [101] is an excellent tool to confirm PK-resistant PrP. However, compared to immunohistochemistry it offers less possibility to analyze subcellular distribution.

#### 4.2.2. Aspects of Classification

There are two aspects of the classification of prion diseases. The first is based on the aetiology; idiopathic, acquired and genetic forms are distinguished. Clinicopathological phenotypes include CJD (sporadic, iatrogenic, variant, or genetic), kuru, GSS, and familial or sporadic fatal insomnia (Table 1) [102]. The second is based on immunostaining for disease-associated PrP, biochemical examination the size of the PK-resistant core of the abnormal prion protein, PrP^Sc^ (*i.e.*, type 1 migrating at 21 kDa and type 2 at 19 kDa), and evaluation of the *PRNP* (mutations and the genotype at the polymorphic codon 129; methionine, M, or valine, V) [102]. According to this, current classification of sporadic CJD recognizes at least six major molecular subtypes and additionally their combinations [34,35,75,103]. The Western blot pattern and morphology of PrP immunoreactivity is used to suggest the BSE-related variant CJD, VPSPr, and certain genetic forms of disease. Differentiation of acquired or genetic forms requires data on the aetiology (*i.e.*, to exclude iatrogenic forms) and sequencing of the *PRNP* together with morphology (*i.e.*, genetic forms such as CJD, FFI or GSS type).

### 4.3. Tauopathies

#### 4.3.1. Overview of Neuropathological Features

For the diagnostic practice antibodies against tau isoforms and different phosphorylation epitopes are used. Hyperphosphorylated tau is the major constituent of neuronal and glial inclusions. Tauopathies are classified as primary or secondary. Based on the cellular distribution of tau pathology, disorders with neuronal (Pick disease, PiD; AD, neurofibrillary tangle (NFT)-dementia or primary age-related tauopathy, PART), mixed neuronal and glial (progressive supranuclear palsy, PSP; corticobasal degeneration, CBD; argyrophilic grain disease, AGD) and glia-predominant forms (globular glial tauopathies, GGT) can be distinguished. Neuropathological classification conforms with different bands and isoforms demonstrated in Western blots of insoluble tau [37,104]. 

Neuronal tau immunoreactivities comprise (1) pretangles; (2) NFTs; (3) Pick bodies; (4) spherical cytoplasmic inclusions; (5) dystrophic neurites; (6) threads; and (7) grains [105]. These are variably argyrophilic or ubiquitin/p62 immunoreactive. For example, pretangles are thought to represent an early stage of development into ubiquitinated and argyrophilic NFTs [106]. 

Astrocytes show a variety of tau immunoreactivities, often labelled with different terminologies. Four major types are seen in primary tauopathies. Differentiation of tufted astrocyte (PSP) from astrocytic plaque (CBD) is the most relevant [105]. Globular astroglial inclusions (GAI) are seen in GGT [107,108]. The term ramified astrocyte, referring to cells with eccentric nuclei and Gallyas-positive branched thick processes, was used to describe tau-positive astrocytes in PiD [109]. In addition, two further morphologies were recently defined in the frame of aging-related tau astrogliopathy (ARTAG) [110]. ARTAG includes thorn-shaped astrocytes (TSA) in diverse locations (e.g., subpial, subependymal, perivascular, white and gray matter) and granular/fuzzy astrocytes (GFA) showing diffuse fine granular tau immunopositivity along astrocytic processes with dense tau immunoreactivity of the soma [110]. Most of the astrocytic tau immunoreactive structures are 4R positive, however, ramified astrocytes in PiD and protoplasmic astrocytes in PSP may show 3R-tau immunopositivity [111,112]. Moreover, tau phosphorylation sites, conformational modifications, tau truncation, and ubiquitination in astrocytes differ between various types of tauopathies [111]. 

Tau (for example using the AT8 antibody) immunoreactivity in oligodendrocytes comprises of coiled bodies and globular oligodendroglial inclusions (GOIs) [107,108]*.* GOIs are characteristic for GGT. While coiled bodies are consistently 4R-tau immunoreactive, occasional GOIs in GGTs may contain 3R-tau as well [111].

These tau pathologies can be observed in a wide range of neurodegenerative conditions, interpreted as “secondary” phenomena; including genetic disorders, where the primary molecular pathology involves different proteins [105,113]. Furthermore, recent studies reported unexpected tau pathologies in disorders that were not initially related to them. For example, increased 4R/3R tau isoform ratio and total tau protein content and increased numbers of rod-like tau deposits that partially or totally spanned the neuronal nuclear space detectable by 4R-specific antibody (RD4) and with antibodies that recognize total tau (Tau5 and HT-7) have been reported in HD brains [114].

There is a paucity of data on the anatomical distribution of distinctly phosphorylated tau (“phospho-tau pattern”) in brains with NDDs. Potentially, the recently described biochemical alterations, such as the lack of acetylated tau in the inclusions in AGD [115], or differences in phosphorylation of tau epitopes in tangles [38] or of C-terminal fragments and trypsin-resistant band patterns in tauopathies [39] might be developed to allow better grouping.

#### 4.3.2. Aspects of Classification

Despite the many forms of biochemical modifications of tau protein (Table 2) [31], current classification of tauopathies focus on the differentiation of 3R and 4R and mixed 3R + 4R types, together with the banding patterns of sarkosyl-insoluble tau, and for further subclassification the morphology and anatomical distribution of neuronal and glial (astro- and oligodendroglial) immunoreactivities are considered. Similar concepts are implemented to characterize tau pathologies, which are associated with disorders of diverse aetiologies [105,113]. Importantly, for some tauopathies it has been suggested that the pathological alterations follow a stereotypic pattern. Indeed, Saito and colleagues [116] reported three stages (expanded to four by Ferrer *et al.* [117]) of the pathology of AGD; furthermore, Williams *et al.* suggested a grading system for PSP, which reflects a progressive involvement of different anatomical areas [118]. Recently, it has been suggested that in PiD tau neuropathology might originate in limbic/paralimbic cortices [119]; this was also noted in a smaller cohort of a GGT subtype where the white matter pathology was shown to involve limbic areas early [120].

### 4.4. α-Synucleinopathies

#### 4.4.1. Overview of Neuropathological Features

α-Synucleinopathies may be neuron- or glia-predominant [121]. Dementia with Lewy bodies (DLB) and PD shows predominance of intraneuronal cytoplasmic and neuritic deposits (cortical and brainstem type Lewy bodies and Lewy neurites), whereas multiple system atrophy (MSA) is dominated by glial cytoplasmic inclusions (Papp-Lantos bodies). In spite of studies, which suggest that the biochemical pattern of α-synuclein may differ in disorders with Lewy bodies and MSA [122,123], currently, only clinical and morphological aspects are considered for the classification of α-synucleinopathies. 

For PD and DLB only Lewy bodies and neurites are evaluated for diagnostic classifications. Some studies suggest that, in addition to α-synuclein, further protein deposits in the striatum, in particular Aβ and less tau, may be a morphologic substrate for the distinction between DLB and PD with dementia [124,125]; these are currently mostly distinguished upon clinical criteria. Lewy bodies can be detected using traditional stainings; however, immunohistochemistry reveals much more pathological structures. Mapping of further α-synuclein immunoreactivities as a tool for further subclassification is hindered by the fact that different studies used distinct anti-α-synuclein antibodies. Most of the antibodies, which are generated against different epitopes of the protein, cross-react with the monomer, physiological form of α-synuclein, and show a synaptic like staining in non-diseased brains [126,127,128,129]. Additionally, various epitope-retrieval methods are used, which also need to be harmonized [126,127]. Thus, the type of immunoreactivities and the amount of immunolabelled intracellular and neuritic structures vary significantly between antibodies and pre-treatments used. Although the PK-resistant form of α-synuclein is considered to be disease-associated on a biochemical level [130], using PK as a pre-treatment tool for immunohistochemistry does however show variable immunoreactivities in diseased but also non-diseased brains, depending on the antibody used. A study using PET-blot (similarly as used in prion disease) suggested a synaptic-like pattern to be associated with DLB [131,132]. Interestingly, using immunohistochemical methods, this pattern cannot be clearly detected as a disease-specific phenomenon (*i.e.*, it is seen also in controls) or overlaps with diffuse neuropil staining, and strongly depends on the antibody used [127,128,133]. Indeed, the extremely PK-resistant species revealed are likely to represent aggregates with high beta-sheet content that occur late in the misfolding process [130]. Interestingly, proximity ligation assay [134] and immunohistochemical studies with an antibody reacting with α-synuclein oligomers [128] show that α-synuclein oligomers in the human brain display a distinct PK resistance as compared to monomers of fibrils. Care must be taken, since Mori and colleagues found strong synaptic and unexpected glial immunoreactivity using PK-treatment of the brain samples in non-diseased-individuals [135]. In summary, PK-treatment might differentially affect detectability of oligomer, monomer, or fibrillar forms of α-synuclein.

A recent study suggested that by using an α-synuclein proximity ligation assay further pathological alterations in neurons could be seen in Lewy body disorders [134]. The authors reported that the method used detected so-called pale bodies in neurons more sensitively and found oligomeric α-synuclein pathology in neuroanatomical areas ahead of the α-synuclein immunoreactivities seen with the α-synuclein antibody used for immunohistochemistry in their study. It must be noted, however, that compared to many antibodies that are used in different studies, which cross-react with the monomeric α-synuclein, antibodies, which detect disease-specific forms such as phospho-α-synuclein at Ser129 or oligomers, also reveal more prominent α-synuclein pathology [128,129,136,137]. These include small thin neurites and dot-like profiles in the neuropil, bundles of threads, and variably also glial α-synuclein pathology. Furthermore, many antibodies label fine dots in neurons, which do not react with p62 or ubiquitin, and are considered to be pre-aggregates, analogously to pretangles in tauopathies [138]. Ultrastructural examinations using a highly disease-specific antibody demonstrated that, similarly to that seen in human prion diseases, these are associated with the ELS [136] supporting the concept of prion-like spreading and uptake of pathological α-synuclein by neurons in the human brain. Importantly, the role of the ELS in the pathogenesis of Lewy body diseases have been supported by genetic studies [139]. Therefore, as already suggested for prion diseases [93], PD could also be interpreted as a form of storage disorder where the overloading of the ELS leads to accumulation of a protein [139].

Recent findings indicate that changes of phosphorylated α-synuclein levels in cingulate and temporal cortices coincided with the early appearance of the Lewy body pathology in olfactory bulb and substantia nigra, even though this was lacking in the cortical region [140]. Moreover, properties of Ser129 phosphorylated α-synuclein changes with progression of Lewy-type histopathology in human brains [141]. The observation that α-synuclein phosphorylation and α-synuclein truncation are normal events in the adult human brain emphasize the need for cautious and harmonized evaluation of α-synuclein-related pathologies, even if it does not necessarily contradict a putative role of phosphorylated α-synuclein Ser129 or truncated α-synuclein in Lewy body formation [142]. 

Further, in Lewy body diseases relatively underappreciated, α-synuclein immunoreactivities can be seen in astrocytes and less frequently in oligodendrocytes. Interestingly, astrocytic immunoreactivity can be seen when using an antibody that recognizes the NAC region of the α-synuclein molecule and formic acid pre-treatment [143], but they are also seen with a disease-specific (including oligomers) antibody [136]. It has been suggested that the amount of astrocytic α-synuclein immunoreacitivity correlates with the fine neuritic and dot-like immunoreactivities but not with the neuronal intracytoplasmatic ones [136]. Interestingly, astrocytes also take-up α-synuclein [136]. The role of astroglia is emphasized in α-synucleinopathies [144,145]. In spite of studies on the relation of microgliosis to α-synuclein pathology and experimental observations on the role of microglia cells in the pathogenesis of α-synucleinopathies [146], there is a lack of unequivocal demonstration of significant deposition of pathological α-synuclein in microglia in the human diseased-brain. Finally, further α-synuclein immunoreactive deposits have been documented, exemplified by deposits in the ependyma, perivascular cells, cranial nerves, retina, but also in peripheral organs and skin [136,147,148,149,150,151,152,153,154,155,156,157,158,159]. These studies help us to understand how pathological α-synuclein potentially reaches body fluids and provide the possibility to develop biopsies as tissue-based biomarkers [158,160]. Again, care must be taken as to how the immunoreactivities are interpreted, since most of the commercially available antibodies cross-react with the monomer physiological form of α-synuclein, which can be then detected in epithelial cells. Regarding phospho-α-synuclein cross-reaction with neurofilament proteins or stress induced-phosphorylation of α-synuclein must not be interpreted as disease-specific alteration [161].

In cases with MSA, the predominant pathology comprise oligodendroglial Papp-Lantos inclusions, furthermore, neuronal cytoplasmic and nuclear inclusions are described. Recent findings report more extensive neuronal α-synuclein pathology [162]. α-Synuclein immunoreactivity has been described in subpial and periventricular astrocytes in MSA of long duration [163] and also in Schwann cells [164]. Skin biopsy may show differences when compared to samples from PD patients [156,159]. 

#### 4.4.2. Aspects of Classification

Classification of diseases with Lewy bodies requires documentation of the clinical data (movement disorder or cognitive decline as early symptom) and the distribution of Lewy body and Lewy neurite pathology. Cluster analysis suggests that there are subtypes of PD [165], furthermore, DLB might be different from PD and PD with dementia [166,167]. Although there are differences in the distribution of α-synuclein pathology and concomitant protein depositions, there are however no biochemical or morphological features, which allow unequivocal distinction of potential molecular subtypes of Lewy body disorders. 

Currently, two neuropathological approaches are used. One is the staging of α-synuclein pathology according to Braak, based on the involvement of the medulla oblongata (stage 1), pons (2), mesencephalon (3), in particular, the substantia nigra), limbic areas (4), and neocortical areas (5) and (6) [168]. Another classification originates from Kosaka [169] and was implemented in the diagnostic criteria for DLB (*i.e.*, brainstem, limbic, and neocortical types) [170]. A further form is represented by Lewy bodies restricted to the amygdala [171,172]. Since the olfactory bulb is a region affected early, there are suggestions for further classification; the so called unified staging system for Lewy body disorders suggests classification of cases into one of the following stages: I. Olfactory Bulb Only; IIa Brainstem Predominant; IIb Limbic Predominant; III Brainstem and Limbic; IV Neocortical [173]. It must be noted that not all cases strictly follow the Braak stages supporting the notion that there are subtypes of diseases with Lewy bodies where α-synuclein pathology is generated “spontaneously” in a specific region [174]. As with tau pathologies, Lewy related pathologies can be seen in a wide range of disorders with diverse aetiologies [166,175,176]. It seems that the distribution of α-synuclein pathology and the presence of further protein deposits (e.g., Aβ) is an important aspect for the classification of clinically different Lewy body disorders [166]. Importantly, there are further immunoreactivity patterns described in Lewy body diseases (see Section 4.4.1.), depending on the antibody used. Meticulous evaluation of these might help to define subtypes. Currently, biochemical markers (summarized in Table 2) are not implemented in further subtyping of PD or DLB.

Similarly to MSA, clinical subtypes are defined [177], nevertheless, these cannot be clearly translated into biochemical or morphological differences. For the diagnosis of MSA the presence of oligodendroglial inclusions (Papp-Lantos bodies) is sufficient [178]. The distribution of glial inclusions follows striatonigral or olivopontocerebellar predominance in a subset of cases corresponding to the clinical classification of MSA-P (parkinsonism dominant) and MSA-C (cerebellar symptom predominant) [177]. Recently, a further type has been described with FTLD [179,180]; further studies are needed to determine whether these are associated with any recognizable molecular signature. 

### 4.5. TDP-43 Proteinopathies

#### 4.5.1. Overview of Neuropathological Features

TDP-43 is a major component of the ubiquitin-positive inclusions that characterize ALS and a common form of FTLD. In addition to sporadic forms, several mutations in different genes, including the *C9orf72* (most frequent), granulin (*GRN*), valosin-containing protein (*VCP*), *TARDBP*, *SQSTM1 *(sequestome), *DCTN1* (dynactin), and *OPTN *(optineurin) have been reported to be associated with TDP-43 proteinopathy [181,182,183,184,185,186,187,188,189,190]. The spectrum of TDP-43 immunoreactive structures includes neuronal cytoplasmic inclusions (NCIs), dystrophic neurites (DNs), neuronal intranuclear inclusions (NIIs), and glial cytoplasmic inclusions (GCIs). Co-localization studies with the oligodendroglia marker TPPP/p25 demonstrated that the latter are mostly oligodendroglial inclusions [191]. The oligodendroglial inclusions may be thin coiled body-like, but also globular morphologies have been described [183,192,193,194,195]. There are further pathological structures, which are more specifically described, like skein-like and spherical inclusions in susceptible neurons in ALS, or the diffuse or granular, or dash-like aggregates, which are dispersed throughout the neuronal somatodendritic domain and extend into the proximal portions of the axon, considered also as “pre-inclusions” since they are not ubiquitinated or p62 positive [104,196,197,198,199,200]. Indeed, as a “next step”, a small number of the neurons display a clustering of the aggregated material, which forms thick net-like and spherical inclusions that are ubiquitinated and p62-immunopositive [197]. Our study using double immunolabelling and immunogold electron microscopy excluded colocalization of phosphorylated (p)TDP-43 with the ELS [201], later supported by findings of others using an oligomer TDP-43 antibody [202]. These data somehow contrast the observations on neuronal cytopathology in AD, prion diseases or PD, where the disease-specific proteins are processed within endosomes, and therefore a distinct role of the ELS in motor neuron diseases has been suggested [93,94,136,201,202]. 

TDP-43 pathology frequently associates with other disorders, including AD, DLB, and frequently with a hippocampal sclerosis (HS; a disorder affecting about 10% of individuals over the age of 85 years [203]) of ageing (in around 90% of cases), but also chronic traumatic encephalopathy; some of these associate with genetic risk factors [19,203,204,205,206,207]. The cellular distribution may resemble that observed in FTLD-TDP with only rare neuronal nuclear inclusions. There are also distinct features, like neurofibrillary tangle-like [208] or grain-like structures [209], or clusters of threads resembling astrocytic plaques in CBD [210]. A recent study suggested that TDP-43 deposition in AD follows a stereotypic progression in five distinct topographic stages, supported by correlations with neuroimaging and clinical features [211,212]: stage I involves the amygdala; stage II spreads into entorhinal cortex and subiculum; stage III shows involvement of the dentate gyrus and occipitotemporal cortex; stage IV shows further spread into inferior temporal cortex; and finally stage V involves the frontal cortex and basal ganglia. Hatanpaa *et al.* reported that TDP-43-positive fine neurites in the pyramidal layer of the hippocampus might be a precursor lesion of HS [195]. A study on TDP-43 pathology in HS in DLB confirmed this suggesting that HS in LBD shares underlying disease mechanisms with FTLD-TDP [213]. 

#### 4.5.2. Aspects of Classification

Originally, subtypes of FTLD-U (where U represent ubiquitin-only; later designated as FTLD-TDP) were distinguished using findings of ubiquitin immunohistochemistry, and two major classifications were in existence [214,215]. Recently a harmonized classification system was proposed, which distinguishes four subtypes (A–D) based on the predominance and distribution of neuritic (DN) and neuronal cytoplasmic (NCI) or intranuclear (NII) inclusions [186]. Briefly, type A is characterized by numerous NCIs, and variable numbers of DNs concentrated in layer 2 of affected cortices; type B is predominated by numerous NCIs in both the superficial and deeper cortical layers, but also occasional DNs can be seen; type C is associated with long DNs all cortical layers; finally, type D is characterized by numerous NIIs, DNs and infrequent NCIs. Interestingly, gene mutations can associate with specific types, exemplified by the high specificity of type D to *VCP* mutations; *GRN* is mostly type A and *C9orf72* mostly with type B or A [184,216]. Importantly, however, cases carrying a *C9orf72* expansion repeat mutation contain additional p62-positive, TDP-43-negative neuronal cytoplasmic inclusions, which are composed of dipeptide repeat proteins (translated from the *C9orf72* expansion repeats) [184].

A recent study using pTDP-43 immunohistochemistry reported patterns of disease progression in bvFTD cases [217]. Accordingly, pTDP-43 pathology started in the orbital gyri, gyrus rectus, and amygdala, later involved the middle frontal and anterior cingulate gyrus as well as in anteromedial temporal lobe areas, the superior and medial temporal gyri, striatum, red nucleus, thalamus, and precerebellar nuclei [217]. More advanced cases showed involvement of the motor cortex, bulbar somatomotor neurons, and the anterior horn of the spinal cord, whereas cases with the highest burden of pathology showed pTDP-43 pathology in the visual cortex as well [217]. Four stages were also recognized for ALS related pTDP-43 pathology [192]: ALS cases with the lowest burden of pTDP-43 pathology showed involvement of the agranular motor cortex, brainstem motor nuclei of cranial nerves V, VII, and X-XII, and spinal cord α-motoneurons. Increasing burdens of pathology showed affection of the prefrontal neocortex, precerebellar nuclei, brainstem reticular formation, and the red nucleus. Later pTDP-43 pathology involved the prefrontal and then postcentral neocortex and striatum [192]. Finally, cases with the greatest burden of pTDP-43 lesions showed involvement of the anteromedial portions of the temporal lobe, including the hippocampus [192]. 

In summary, the most important aspect for disease-classification is the anatomical distribution and morphology of (phosphorylated)-TDP-43 deposits and neuronal loss complemented by genetic examinations. Currently there are no biochemical properties, which are used for the subclassification of TDP-43 proteinopathies.

### 4.6. FUS-Proteinopathies

FUS pathology characterizes familial ALS cases and a rare group of diseases with FTLD. Sporadic disorders of FTLD, such as basophilic inclusion body disease (BIBD), atypical FTLD-U (aFTLD-U), and neuronal intermediate filament inclusion disease (NIFID), feature neuronal (cytoplasmic/nuclear) and glial cytoplasmic inclusions immunoreactive for FUS, thus establishing a new category (FUS/FET proteinopathies) [21,41,218,219]. Immunoreactivity for further FET proteins is helpful to distinguish sporadic and FUS-gene mutation related cases [21]. The inclusion types and their regional distribution in FTLD are sufficient to distinguish them in the majority of cases. In addition, postsynaptic localization of FUS was also considered to be important [220], but was not translated into disease-classification. In summary, classification of FUS/FET proteinopathies is based only on morphological criteria; however, distinct stainings with FET proteins can be helpful also when a genetic origin is considered [21].

### 4.7. Rare Forms of Hereditary Neurodegenerative Conditions with Protein Deposition

There are more forms of genetic NDDs with abnormal protein inclusions, comprising proteins encoded by genes linked to neurological trinucleotide repeat disorders. Their basis is the expansion of unstable trinucleotide repeats that account for disorders, ranging from childhood disorders to late onset diseases such as inherited ataxias and HD. Intraneuronal, intranuclear and neuritic (axonal), mostly ubiquitinated and p62 positive inclusions are detected in HD [221,222]. Based on the worsening and topographical spread of the degenerative process of the striatum, a neuropathological grading system has been developed [223]. One large group of disorders represent SCAs and dentatorubro-pallidoluysian atrophy (DRPLA), which show heterogeneous clinical presentations and have distinct genetic backgrounds with several underlying mutational mechanisms [14]. With regard to the non-polyglutamine SCAs less information is available, because they are rare and/or have only recently been identified as clinical entities [14]. The aggregation of gene products into neuronal intranuclear inclusions is an established feature of SCA1, SCA3 and SCA7, while in SCA2 and SCA6 primarily neuronal cytoplasmic aggregations are reported [14], while ubiquitinated glial inclusions are seldom described [224]. Spinal and bulbar muscular atrophy (SBMA), also known as Kennedy’s disease, is a slowly progressive motor neuronopathy, which is X-linked and is caused by an expansion of the CAG repeat in the androgen receptor (AR) gene [225] and fragile X-associated tremor and ataxia syndrome (FXTAS), which is associated with CGG repeats in the *FMR1* gene, also show neuronal intranuclear inclusions [226]. In contrast, the GAA expansion in Friedreich ataxia does not associate with inclusion body formation of the gene product [227]. 

A further rare inherited disorder is associated with the serine protease inhibitor neuroserpin; the encoding gene is mapped to chromosome 3q26 [228,229]. Neuroserpin is present in the neuronal cytoplasm and processes but not in the nucleus [228,229,230,231,232]. In ferritin-related NDDs, the molecular genetic defect resides in the ferritin light polypeptide gene located on chromosome 19 [233]. Ferritin deposits in the nucleus and cytoplasm of neurons and glia, but also in endothelial cells, cells of the vascular adventitia, choroid plexus, and leptomeningeal cells, and peripheral organs [233].

Other diseases show similarities to the pathology of AD including CAA; however, the protein composition is different. These are genetically and biochemically diverse disorders, which uniformly show the amyloid fibrils deposited in the walls of small to medium-sized, mostly arterial blood vessels, but also in capillaries of the CNS parenchyma and leptomeninges [234]. The deposited proteins include Aβ, transthyretin, gelsolin, cystatin, PrP, and BRI2 protein [234]. The neuropathological hallmarks of familial British dementia resembles AD, including parenchymal ABri amyloid and preamyloid plaques, widespread ABri-CAA and neurofibrillary tangle pathology, while in familial Danish dementia, the parenchymal lesions are primarily of preamyloid nature and there is also frequent co-deposition of Aβ [234]. 

Some further disorders exhibit inclusions, which are immunoreactive only for the ubiquitin-proteasome system (*i.e.*, FTLD-UPS) or a variety of proteins like in intranuclear inclusion body disease (INIBD). INIBD is a neurodegenerative disorder mostly presenting infantile or juvenile fatal condition, while in adults it is thought to be very rare [235]. It is characterized by eosinophilic nuclear inclusions that are immunoreactive for ubiquitin and related proteins in neuronal but also in glial cells and in peripheral organs [236,237]. Interestingly, these inclusions are labelled by antibodies against neurodegeneration-related proteins, such as optineurin or FUS [238,239]. Although INIBD is rare, it has been reported in community based studies [18] and can also be associated with other rare NDDs like CJD [240].

Finally, there are diseases where no specific inclusion can be detected (e.g., FTLD-ni; no inclusions) [185] in spite the presence of system-related neurodegeneration. Some conditions like those associated with brain iron accumulation, postencephalitic disorders, head trauma, or others observed in restricted geographical areas (e.g., parkinsonism-dementia complex of Guam) associate with various spectra of proteinopathy lesions reminiscent of, or overlapping with, the features of major NDD disease entities. 

## 5. Synthesis of Biochemistry, Genetics, and Morphology

Biochemical examinations have shown several differences between the proteins in physiological conditions and in disease. These modifications may affect the solubility, and capability of fibril formation, or toxicity of these proteins. Some of these overlap between proteins, such as formation of β-sheet structure, proteinase-resistant cores, oligomerization, and phosphorylation. However, from a wide spectrum of biochemical modifications currently only few have been translated into the diagnostic practice: PK-resistance for PrP, phosphorylation for tau, α-synuclein, and TDP-43, isoform differences for tau, and cleavage products for Aβ. Detection of truncated products of tau and α-synuclein, and oligomers of Aβ, PrP, tau, or α-synuclein is beginning to be implemented. However, the spatial and temporal distribution of the different pathologic protein species has not yet been elucidated in details. Most importantly, we need biochemical markers to define clinically relevant subtypes of proteinopathies.

Morphology and anatomical distribution of pathological alterations still predominates the classification of AD, α-synucleinopathies, TDP-43 and FUS/FET proteinopathies (Table 3). Gene mutations also associate with the proteinopathies discussed here. Thus, a genetic background should be considered in all of these; this is out of the scope of this review. Examination of influential gene polymorphisms, together with biochemical aspects, has been implemented into the subclassification strategy only for prion diseases. For tauopathies, in addition to morphology, biochemical aspects are considered (isoforms). Regarding the distribution of pathological protein deposits, there are overlapping features seen in different proteinopathies, such as (1) differentiation based on extra- (Aβ, PrP) or intracellular (tau, α-synuclein, TDP-43, FUS) predominance of protein deposits; (2) distinguishing neuronal-predominant, mixed neuronal-glial, and glial predominant forms of proteinopathies; (3) evaluation of stages and phases is implemented in a growing number of disorders (PD, AGD, AD, TDP-43 proteinopathies); (4) presence of intracellular protein deposits representing early steps (*i.e.*, “pre-aggregates”) and ubiquitinated structures representing later steps of intracellular protein pathologies. 

**Table 3:** Molecular pathological features that are currently considered for subtyping and those, which are disease-specific but not yet implemented for classifications. * Depends also on the method (*i.e.*, immunohistochemistry or paraffin-embedded-tissue (PET)-blot method).

Disease	Molecular Pathological Features	
Disease Group	Currently Used for Subtyping	Disease-Specific but Not (yet) Crucial for Subtyping
**AD-related pathology**	Anatomical distribution of neuronal tau pathology	Truncated Aβ species
	Anatomical distribution of extracellular Aβ deposits	Pyroglutamate modifications
	Presence and ditribution of CAA	Phosphorylation patterns of Aβ and tau
	/	Subtyping based on predominance of NFT
**Prion disease**	Morphology of PrP deposition	Oligomer forms
	Glycosylation pattern and electrophoretic mobility of PK-resistant PrP (only WB)	/
	Codon 129 polymorphism	/
	Aetiology if known	/
**Tauopathies**	Morphology of neuronal or glial protein deposits	Detecting phosphorylation epitopes
	Distinguishing 3R and 4R isoforms	Acetylation
	Anatomical distribution of protein deposits	Truncated forms (*i.e.,* C-terminal)
	/	Trypsin-resistant band patterns
	/	Oligomer forms
**α-Synucleinopathies**	Morphology of neuronal or glial protein deposits	Phosphorylation
	Anatomical distribution of protein deposits	Nitration
	/	Oligomer forms
	/	Predominance of soluble/insoluble form
	/	Truncated forms
	/	Detection of PK-resistant form *
**TDP-43 proteinopathies**	Morphology and subcellular distribution of protein deposits in neurons	Phosphorylation
	Anatomical distribution of protein deposits	C-terminal fragments
	/	Glial inclusions
**FUS proteinopathies**	Morphology, subcellular and anatomical distribution of protein deposits	Different immunoreactivity for FET proteins
	/	Glial inclusions

**Figure 2:**
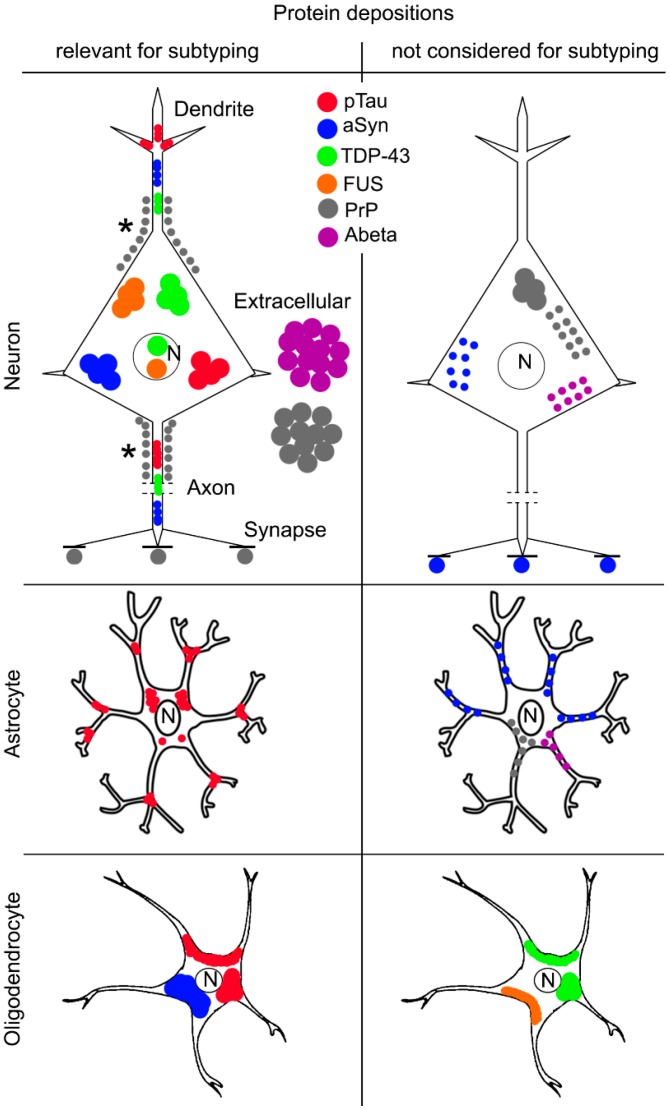
Overview of cellular vulnerability in the most frequent neurodegenerative proteinopathies. N: nucleus. Asterisk (*) for PrP indicates periaxonal or perineuronal. Since the drawing of the full extent of an axon would be out of the image the lines (---) in the axons indicate discontinuation (at the middle segment of the axon) of the drawing. Synapses are indicated by short black and bold lines (-).

**Figure 3:**
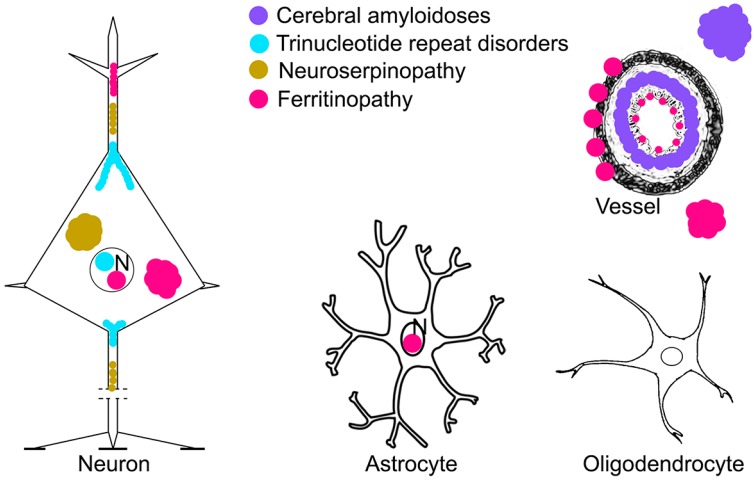
Cellular vulnerability patterns in rare hereditary forms of neurodegenerative diseases. N: nucleus. Since the drawing of the full extent of an axon would be out of the image the lines (---) in the axons indicate discontinuation (at the middle segment of the axon) of the drawing. Synapses are indicated by short black and bold lines (-).

It is interesting to see how different proteins distribute in cells and subcellular structures to understand overlaps in the pathogenesis of different disorders (Figure 2 and Figure 3). 

**Figure 4:**
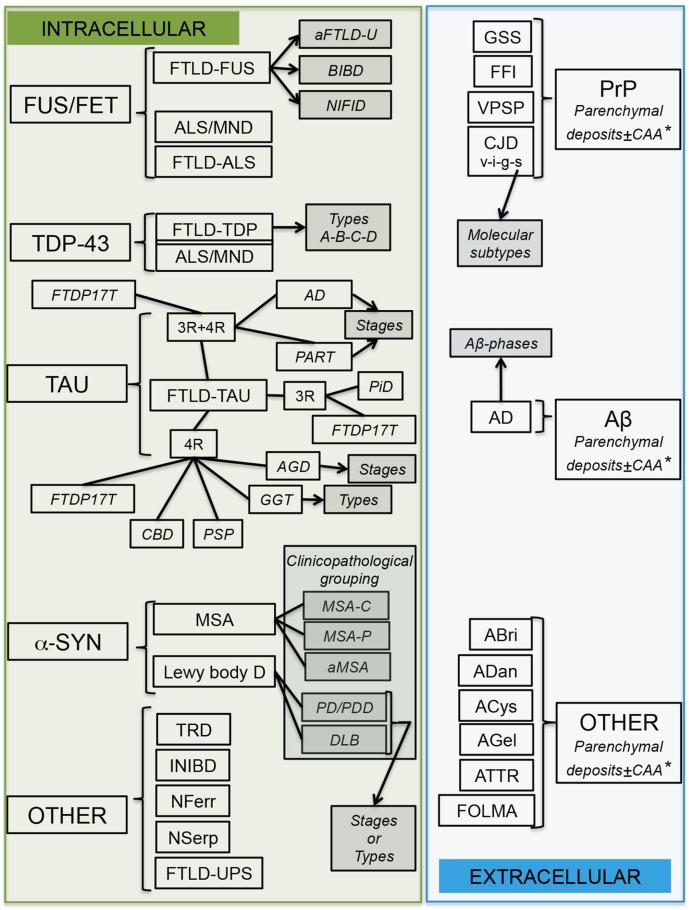
Algorithm for the classification of neurodegenerative proteinopathies, Abbreviations: Abri and ADan: amyloidoses related to familial British dementia and familial Danish dementia; ACys: amyloidosis related to Cystatin C amyloid; AD: Alzheimer disease; AGD: Argyrophilic grain disease; AGel: amyloidosis related to Gelsolin amyloid; ATTR: amyloidosis associated with transthyretin amyloid; ALS: amyotrophic lateral sclerosis; BIBD: Basophilic inclusion body disease; CAA: sporadic cerebral amyloid angiopathy; CBD: Corticobasal degeneration; CJD: Creutzfeldt-Jakob disease; DLB: Dementia with Lewy bodies; FFI: fatal familial insomnia; FOLMA: familial oculoleptomeningeal amyloidosis; FTLD: frontotemporal lobar degeneration; aFTLD-U: atypical FTLD with ubiquitinated inclusions; FTLD-UPS: FTLD with inclusions immunoreactive only for the components of the ubiquitine proteasome system; FTDP-17T: Frontotemporal dementia and parkinsonism linked to chromosome 17 caused by mutations in the *MAPT* (tau) gene; GGT: globular glial tauopathies; GSS: Gerstmann-Sträussler-Scheinker disease; INIBD: intranuclear inclusion body diseases; MND: Motor neuron disease; MSA: multiple system atrophy (C: cerebellar, P: Parkinsonism, aMSA: atypical MSA); NFerr: neuroferritinopathy; NIFID: Neurofilament intermediate filament inclusion disease; NSerp: neuroserpinopathy; PART: primary age-related tauopathy; PD: Parkinson disease; PDD: PD with dementia; PiD: Pick disease; PSP: Progressive supranuclear palsy; TRD: trinucleotide repeat expansion disorder: refers to genetic disorder and associated with different proteins; VPSP: variably proteinase sensitive prionopathy. For CJD, v: indicates variant, s: sporadic, i: iatrogenic, and g: genetic CJD. Kuru is not indicated in this figure. Note that overlap between FTLD-TDP and ALS/MND indicates combined phenotypes (FTLD-ALS/MND). * indicates that PrP-CAA is very rare; for Aβ, CAA is frequent, for other amyloidoses, CAA is more frequent than parenchymal deposits. Note that FTLD-ni is not indicated here, since no proteinopathy is associated with it. ± indicates with or without. Green and blue coloured box indicates intra-, or extracellular proteins; gray box indicates clinical and/or pathological subtypes; arrows point to subtyping based on pathological aspects.

These aspects associate with different biochemical modifications of a specific protein and further, yet under recognized changes of the cells (*i.e.*, lipid, metabolomics), which could have implications for defining early (presymaptomatic) and late (symptomatic for subtyping for prognostics) biomarkers of diseases. An overview of classification is presented in Figure 4.

Another aspect (out of the scope of this review) is the frequent occurrence of concomitant proteinopathies, which leads to difficulties of disease subtyping. This means that in addition to the hallmark lesions of a NDD entity, further pathological alterations can be observed in the same brain. Deposition of multiple neurodegeneration-related proteins together with non-neurodegenerative pathologies (vascular, metabolic, *etc.*), is a frequent event [15,241]. A recent community-based neuropathology study is a proof of this concept, since it revealed a large variety of proteinopathies with different combinations [18] reflecting the biological variability and arguing against simplified classifications. These findings might have implications for (1) therapy strategies aiming to target single pathological proteins in the brains of elderly individuals with dementia; furthermore (2) for the stratification of patients for biomarker or genomic research. Understanding the concept of “lowering the threshold” for a clinical symptom is crucial for the clinical and neuropathological practice. For example, the threshold of cognitive impairment could be reached by a prominent amount of pure AD-related changes but could be reached by the concomitant presence of several distinct neuropathological alterations that themselves alone would be not sufficient to cause dementia [16,18]. Finally, it must be noted that several gene mutations associate also with depositions of different proteins not only those of their gene product (see *βAPP* mutation with α-synuclein pathology or *PRNP* mutations with tau pathology, *etc.*) [242,243].

## 6. Conclusions

The discovery that most of the adult-onset neurodegenerative conditions are associated with depositions of altered proteins changed our approach to NDDs as follows [16]: (1) The altered proteins can be potentially detected in body fluids or visualized in PET imaging; (2) Due to the different subcellular distribution of the pathological proteins, the pathways by which these proteins reach the body fluids might vary; (3) Based on the concept of prion-like spreading, these proteins may be targeted for therapy; (4) Due to the frequent coexistence of proteinopathies [15,18,19], targeting the protein processing systems may help to maintain the healthy homeostasis of cells and to protect the physiological forms of neurodegeneration-associated proteins; (5) The concept of concomitant proteinopathies also implies that detection of a panel of neurodegeneration-related proteins and their modifications (“protein coding of NDDs“) together with other markers, which reflect disease-dynamics in body-fluids combined with neuroimaging and analysis of gene variations can lead to personalized diagnosis or better prediction of prognosis [19].

Currently, precise morphology is still an important tool to distinguish disorders with different disease course and prognosis. Currently available antibodies are seldom selective against disease-specific forms, thus pre-treatments are used, which might be interpreted differently. For example, many antibodies also detect the physiological forms. Previous multicentric studies have shown that harmonizing the methods used can considerably improve the comparability of different studies. This strategy should be maintained, since many new antibodies have appeared but need systematic comparisons and studies on reproducibility. 

*Post mortem* microscopically detectable features need to be translated into *in vivo* biochemically distinguishable biomarkers first to better define prognosis and second to better stratify patients for therapy trials. It is still difficult to interpret the reliability of *in vivo* biomarkers without detailed neuropathological examination that includes the description of the, frequently combined, deposition of NDD-associated proteins as well as additional disorders, like vascular pathologies. Therefore, multidisciplinary and longitudinal studies that comprise clinical and neuroimaging follow-up as well as standardized examination of body fluid biomarkers are crucial. 

In conclusion, oversimplification of disease grouping with the aim of developing therapies for as many individuals as possible showing similar clinical features has not led to significant success. Defining novel clusters of patients with NDDs for stratified therapeutic approaches, based on continuous, meticulous, harmonized, and constantly updated clinical, neuroimaging, biochemical, and genetic classifications with permanent neuropathology-based quality control seems to be a better approach in the era of precision medicine. 

## Abbreviations

Aβ: amyloid-β
AβPP: Aβ-precursor protein
AD: Alzheimer disease
aFTLD-U: atypical FTLD-U
AGD: argyrophilic grain disease
ALS: amyotrophic lateral sclerosis
ARTAG: aging-related tau astrogliopathy
BIBD: basophilic inclusion body disease
BSE: bovine spongiform encephalopathy
CBD: corticobasal degeneration
CJD: Creutzfeldt-Jakob disease
CNS: central nervous system
*DCTN1*: dynactin
DLB: dementia with Lewy bodies
DN: dystrophic neurities
EWS: Ewing’s sarcoma RNA-binding protein 1
ELS: endosomal-lysosomal system
FFI: Fatal familial insomnia
FTD: frontotemporal dementia
FTLD: frontotemporal lobar degeneration
FUS: fused in sarcoma
GFA: granular/fuzzy astrocytes
GGT: globular glial tauopathies
GAI: Globular astroglial inclusions
GOI: globular oligodendroglial inclusions
GSS: Gerstmann-Sträussler-Scheinker disease
GRN: granulin
HD: Huntington disease
*INIBD*: intranuclear inclusion body disease
*MAPT*: microtubule associated protein tau
NCI: neuronal cytoplasmic inclusion
NDDs: Neurodegenerative diseases
NFT: neurofibrillary tangle
NIFID: neuronal intermediate filament inclusion disease
NII: neuronal intranuclear inclusion
*OPTN*: optineurin
PART: primary age-related tauopathy
PET-blot: paraffin-embedded-tissue blot
PD: Parkinson disease
PiD: Pick disease
PK: proteinase K
PrP: prion protein
*PRNP*: gene of PrP
PSP: progressive supranuclear palsy
R: repeat
SCA: spinocerebellar ataxia
SOD1: superoxide dismutase
SNCA: α-Synuclein gene
*SQSTM1*: sequestome-1
TAF15: TATA-binding protein-associated factor 15
*TARDBP*: gene of TDP-43
TDP-43: Transactive response (TAR) DNA-binding protein
TSA: thorn-shaped astrocytes
VCP: valosin-containing protein
VPSPr: variably proteinase sensitive prionopathy
